# The Principles and Practice of Obstetric Medicine and Surgery, in Reference to the Process of Parturition

**Published:** 1841-10-01

**Authors:** 


### THE PRINCIPLES AND PRACTICE OF OBSTETRIC MEDICINE AND SURGERY, IN REFERENCE TO THE PROCESS OF PARTURITION. By *F. H. Ramsbotham*, M.D. &c. 8vo. pp. 672. Churchill, 1841.

THIS work first appeared as an *Atlas of Plates*, of which we made favourable mention in our No. of April, 1840. Fortunately for the medical student Dr. Ramsbotham was subsequently induced to extend it into the form of a treatise, and the work, as it now appears, may be considered as one of the most useful treatises, taken together with the very superior illustrative plates, of obstetric medicine and surgery that we possess. In his preface, Dr. R. says, "this branch of physic, indeed, has struggled against far greater difficulties than have beset the general practice of medicine and surgery; for both ignorance and prejudice have lent their aid towards retarding its advancement. On the one hand it has had to contend with the natural prejudices that females themselves must entertain against admitting a person of the opposite sex to undertake the duties required under the trying time of labour; and on the other, with the erroneous belief that parturition, being a natural action, would be accomplished in woman with equal facility and safety as in the brute creation."

The continental universities took the lead in enrolling *midwifery*, as it is called, among their obligatory studies; and most of the British institutions of a like nature have tardily followed in their steps.

As far as the London corporations are concerned, much may be attributed to the exertions of a society established in 1826, under the title of the Obstetric Society of London. This society consisted of about thirty members, embracing, with the exception of two or three, all the then present and late lecturers on obstetric medicine in London, besides a few other practitioners; and the editor of this work acted as honorary secretary. The object of the society was to place the practice of obstetric medicine on a more respectable footing than it had hitherto enjoyed.

We well remember the exertions of the hon. secretary of that society—who, after eulogising the conduct of Sir R. Peel—then at the head of the Home Office, goes on to say—

"All the objects which the society proposed have since been carried into effect, except the change in the constitution of the council of the College of Surgeons; and thus, to the perseverance of a very few members of the profession may justly be attributed the adoption of measures fraught with the highest possible advantage to the community, inasmuch as they tend to enhance the acquirements of the great mass of English practitioners." 3.

Our author insists most pointedly and properly on the necessity of having a perfect knowledge of all the female parts concerned in the generative process to be enabled to understand the mechanism of parturition, and first speaks of the bony structure to which the soft parts are attached, and of the origin of the term pelvis, from its resemblance to a wooden utensil of the bowl form, πέλυξ, used by the Greeks; and described in many of the older anatomical works as the basin.

On the individual bones composing the pelvis we need not here descant, we shall only particularize such parts of each as may be more important to the obstetrician. At the junction between the ilium and sacrum is interposed a piece of fibro-cartilage, about a sixth or eighth of an inch in thickness, so that the bones are separated to that extent; and it is invariably remarked, provided the joint is healthy, that, when the ligaments are cut, and the two bones forcibly wrenched asunder after death, the cartilage adheres to the sacrum, leaving the ilium denuded. In structure it is more like the intervertebral substance than any other tissue of the body: it is arranged in concentric layers, and is softer towards its posterior edge than in the front. The object of this soft elastic pad being situated in this place is evidently to break the shock, and prevent the jarring sensation which must otherwise have been experienced, in the violent actions of the body, such as running and leaping; and it may also act as a cement in glueing these bones together.

The spinous process of the ischium is an object of more intense interest to the obstetrician than its small size would lead us to suppose; because it is sometimes of undue length, or is bent too much inwardly. By such a construction, the capacity of the outlet is materially encroached upon and diminished, and, in a proportionate degree, the passage of the child's head in labour is retarded.

The pubic bones are not in contact at the symphysis; for there is a considerable thickness of the same kind of cartilaginous matter placed between them as is found at the sacro-iliac symphyses. Some anatomists have affirmed that there is a double joint, one on each side of the central cartilage; others, that there is only one; and others, again, that although occasionally an imperfect synovial membrane may be seen, by far most frequently, neither can a cavity be detected, nor any apparatus indicative of the presence of a joint: and this latter seems to be the idea of the best anatomists of the present day. The late Mr. Joshua Brooks says he never saw an anchylosis formed between these two bones.

"The coccygeal joints are of great value in the process of labour. Their mobility much facilitates the exit of the head, by enlarging the outlet of the pelvis in the antero-posterior direction. The increase of space thus gained amounts to an inch or more; for the point of the bone may be bent backwards to a line continuous with the sacrum, or even beyond, so as to form an angle outwards.

Occasionally, indeed, the coxyx becomes anchylosed to the sacrum, and its own joints also are destroyed by a deposition of osseous matter between the separate pieces, so that their mobility is lost, and the bone becomes, as it were, a portion of the sacrum itself. Such a consolidation must offer a considerable impediment to the expulsion of the head, by contracting the pelvic outlet: and this, though a rare, is therefore another cause of lingering labour. It is most ussually met with in women bearing a first child late in life, and those who have been accustomed to sit through the principal part of the day." 20.

When the coccyx is in this state, it will sometimes break; either during the expulsive efforts of natural labour—or from the employment of instruments. Dr. R. says, where the bone broke or the anchylosed joint gave way—no permanent injury ensued. We have now and then witnessed considerable subsequent suffering where the junction of the bones formed an angle.

*Form and Dimensions of the Pelvis.*—When we examine the pelvis in reference to labour, these are of considerable importance to be attended to.

We observe that it is formed on the principle of the double arch, which structure in architecture possesses the greatest possible degree of firmness that can be devised for the quantity of material employed. So that the pelvis combines, to an eminent extent, the qualities of strength and lightness. Obstetrically it is divided into the *brim,* or superior aperture; the *outlet,* or inferior aperture; and the *cavity,* all that is embraced between these two.

The pelvis itself has two axes, one of the brim, which is downwards and backwards, following a direction from the umbilicus to the coxygeal extremity of the spinal column; and the other of the outlet, which is downwards and forwards, from the promontory of the sacrum to the central space between the tuberosities of the ischia: so that a line drawn through the brim, in the direction of the axis of the brim, would cross, at a considerable angle, another line drawn in the direction of the axis of the outlet.

It is of most essential importance to keep strictly in mind the relation which the *two axes of the brim and outlet* bear to each other; when performing the most simple or complicated obstetrical operation from the mere extraction of the placenta to delivery with the forceps.

Separation of the joints of the pelvis during labour may possibly take place in the lower animals, but it is certainly not usually the case in the human subject.

*Fœtal Head.*—Its shape and dimensions are well delineated—and the necessity for learning the situation of the fontanelles and sutures. An accurate knowledge of the form and situation of these fontanelles is of absolute necessity for the successful practice of the obstetric art; for by them we detect the position of the fœtal head in the early stage of labour. The account of the advantages of the peculiar structure of the fœtal head, would be well worth the perusal of the too great admirers of instruments, pages 34, 35.

*Of Deformed Pelvis.*—The want of due capacity sometimes originates in natural formation; thus a woman of short stature, although of tolerable symmetry, might be expected to possess a diminutive pelvis; but this is far from being an universal, or even general remark. Again, the re-union of the bones after fractures will commonly occasion both distortion and contraction of space; but when there exists a deficiency of room to any great extent, the irregularity is mostly dependent on disease of the bones themselves.

"If we look at the head of the child, and the cavity through which it has to traverse, in a mechanical point of view, (which we must do before we can arrive at a correct knowledge of the process of parturition, even in the simplest and most easy state,) we shall immediately perceive that *size*, as regards the head and the pelvis, is entirely a relative term: and that a pelvis preternaturally small, or a head unusually large, will each in practice occasion difficulty in the same degree as they deviate from the standard dimensions; so that it matters little whether the disproportion be the consequence of diseased action or any other cause; provided it exists, to a certain extent, it must necessarily be productive of a protracted struggle." 37.

There are two diseases particularly, through which the pelvis suffers considerable deterioration in size, rachitis or rickets, a disorder of childhood—and mollities ossium or malacostion, one of adult age. In both these affections there is a want of due solidity in the osseous system throughout the body.

"Deformity may be partial or general,—partial when either of the parts, the brim, cavity, or outlet, is simply the subject of derangement,—general, when all these are more or less involved. If the vicious formation be confined to the brim, the diminution in size is almost always produced by the promontory of the sacrum jutting too far forwards, and by this means contracting the conjugate diameter; if to the cavity, by the sacrum being too straight, so that the bone does not possess its due curvature; if to the outlet, by the tuberosities of the ischia approaching too near each other; or by the spinous processes of the same bones being too long, and directed too much inwards; or again, by the joints of the coxyx having become anchylosed, and having thus lost their mobility. Of these irregularities the most frequent is that met with at the brim; the most rare, an undue straightness of the sacrum." 38.

There is a variety of deformity of which Dr. R. has given a plate from the work of M. Moreau, which much resembles the obliquely contracted pelvis described by Professor Naegele, which he says is a pelvis contracted in the direction of one of its oblique diameters, with complete anchylosis of the sacro-iliac symphysis of one side and imperfect formation of the sacrum and os innominatum of that side. This deformity M. Naegele considers to be of congenital malformation, and not the result of disease: but we agree with the reviewer in the British and Foreign Medical Review and Dr. Rigby, that the professor is not quite borne out in his opinion.

On the inutility of pelvimeters we are quite in accordance with our author—and that it is impossible to detect any deformity of the cavity with them—and therefore the hand is the most applicable for the purpose of taking the internal measurement of the pelvis, the mode of performing which he well describes.

It does not necessarily follow that the pelvis should be distorted—even where considerable curvature of the spine exists—and as a general rule it may be observed, that where the curvature of the spine occurred subsequent to the age of puberty, the pelvis is for the most part unaffected; and, on the contrary, where the curvature of the spine took place during infancy, the pelvis is, for the most part, proportionably affected. It is by internal examination alone that a decided opinion can be given of the state of the passage.

A *preternaturally* large pelvis appears to be by no means of advantage—it fails to give the proper support under many circumstances during pregnancy. From the necessarily great influence this formation of the pelvis has on the process of labour, the whole of the chapter descriptive of it is most worthy the attentive study of the practitioner.

The female generative organs are classed in two divisions—external and internal.

The external organs need not detain us long. In speaking of the fossa navicularis or concha, Dr. R. says, it contains within its precincts the clitoris with its prepuce, the nymphæ, the vestibule, and the meatus urinarius. It seems to us better to confine things to their right names and places, and simply say it is formed betwixt the proper orifice of the vagina or (hymen) and fourchette, or joining of the labia at their lower edge.*

On the meatus urinarius, which is a part of essential importance to be well acquainted with, the natural delicacy of females not allowing of an ocular perception of the part, our author is much too superficial—neither does he describe the mode of passing the catheter—which we think ought to be done, as miserable effects have we often witnessed from the want of this knowledge; and he justly observes, "it is infinitely better to expose the patient to the inconvenience of an ocular inspection than to allow the bladder to become over-charged, to the imminent risk of its bursting, or to the no less probable chance of a fistulous orifice being formed between its neck and the vagina."

Of the internal organs—the vagina, the uterus and its appendages, are well described.

*Corpus Luteum.*—"In the ovary of a woman recently pregnant, we observe, besides these vesicles, a vascular spot about the size of a large pea or small bean, containing a central cavity, sometimes empty, at others filled with coagula, the consequence of the late conception. It is somewhat fabiform, of a dull yellow tint, resembling in hue the buffy coat of the blood, and when newly exposed, slightly red. The name CORPUS LUTEUM was given to it by Malpighi, from its colour; it had been previously called by De Graaf CORPUS GLANDULOSUM, for it possesses much of a gland-like appearance. Hunter, indeed, described it as 'tender and friable, like glandular flesh.' Rœderer compares its structure to that of the supra-renal capsules of the fœtus; and Montgomery speaks of it as 'obviously and strikingly glandular.' Corresponding with its situation externally there is observable a distinct cicatrix on the surface of the ovary, indicating the spot through which the fluid contained in the Graafian vesicle has escaped into the Fallopian tube. The aperture has, in some rare instances, been found still pervious, when the conception was very recent." 66.

The corpus luteum will present different appearances according to the length of time that has elapsed since impregnation. The period of its visibility varies also considerably according to circumstances. Montgomery has found the central cavity existing in the sixth month from impregnation; and the corpus luteum distinguishable at the end of five months from mature delivery, but never beyond that time. From the observations of Dr. Paterson, indeed, (Edin. Med. Journal, Jan. 1840,) it would appear that positive evidence of the existence of this body is rarely met with, even three or four months after labour; so that the common idea that this is a permanent structure, and that an examination of the

* See Bell's Anatomy, Vol. III.

ovaries after death will enable us to tell the exact number of children which any woman has borne, from the number of corpora lutea existing in her ovaries, is quite erroneous.

Of the mode of its formation physiologists are not quite agreed. Dr. Ramsbotham coincides in opinion with Drs. Montgomery and Paterson in preference to Professor Baer and Dr. R. Lee, whose paper, in the 22nd Vol. of the Medico-Chirurgical Transactions, is very clear and distinct.

" *False Corpora Lutea.*—The remark of Haller, that '*conception never happens without the production of a corpus luteum,*' has, I believe, never been disputed; but his other proposition, that '*the corpus luteum is never found in virgin animals, but is the effect of impregnation alone,*' has been canvassed very extensively. Some physiologists have supposed that true corpora lutea, or bodies analogous in appearance to them, can be formed in the ovaries of virgins; while others have expressed themselves so vaguely on the subject, as to leave their opinions in great doubt. The possibility of such an occurrence is a question of first-rate importance in many medico-legal investigations; and consequently it is incumbent on every one who touches upon this subject to endeavour to put it in a clear light." 68.

The whole of the evidences on this point are worthy the most attentive perusal. " In advanced life, the ovaries become shrivelled, corrugated on their surface, firmer in their texture, and often contain empty collapsed cysts, with thickened, opake coats, so strong that they can be turned out of their bed entire. These have been mistaken for, and described as, corpora lutea; but after the account already given, it must be evident that such is not the case. There is little doubt that they are Graafian vesicles altered by age."

Although the ovaries are usually described as appendages to the uterus, the uterus ought in truth to be considered as an appendage to them, for they are the most essential organs in the function of generation. The uterus may be diseased to a great extent, and yet the woman may be fruitful; but if both these glands are much altered in structure, barrenness necessarily ensues. An ovary, indeed, or something analogous to it, is found throughout the whole of the sexual genera of both animals and plants.

For the uterus, with its well-delineated nerves, blood-vessels, &c., of which we may speak hereafter, we must refer to the work itself.

The muscles within the pelvis deserve notice; for, by being pressed on during the escape of the child's head, they are sometimes strained, and pain is experienced in moving the thigh, and in evacuating the rectum, for some days subsequent to labour.

The Fallopian tubes, through which the ovum after impregnation passes into the uterine cavity, are covered externally by the peritoneum, possess a middle coat of muscular fibres which run longitudinally, transversely and obliquely (?), and an internal mucous coat, a continuation of the mucous membrane lining the uterus. It is the only instance in the body where a mucous membrane and a serous membrane join by continuation of structure, the only example of mucous membrane terminating in a shut cavity.

*Analogy between the Genital Organs in the two Sexes.*—Although the organs of generation appear to be widely different in the two sexes, and indeed give them their distinctive characters; yet there is seen, on closely comparing them, a great similarity, not only in function, but even in formation; so that we cannot withhold our belief that they have both been fashioned on a common model. In many instances the confusion arising from this similitude is so remarkable, that it is difficult to decide, particularly in infancy, to which sex the individual belongs.

### OF THE GRAVID UTERUS.

When we compare the unimpregnated with the gravid uterus at the end of gestation, we should be inclined to doubt—from the extraordinary alteration that has taken place during pregnancy—whether in reality they were not two perfectly distinct organs. We observe an amazing difference, indeed, in every essential attribute, particularly in *form, size, situation, texture, power,* and *contents.*

The alteration in SIZE is most remarkable; the virgin uterus measures not more than three inches in length, and two in breadth; when labour is near at hand, it is about thirteen inches long, and eight or nine across.

The TEXTURE of the unimpregnated uterus is close, tough, firm, and inelastic; the structure of the organ when gravid is loose, spongy, and distensible, capable of being drawn out to a considerable extent between the fingers without laceration of its substance. The looseness of its texture depends chiefly on the enormously increased size which its vessels have acquired during the development of the organ.

We believe that a great deal of misconception exists as to the thickness of the gravid uterus in very spare women. The limbs of the fœtus may be grasped with the hand through the abdominal parietes and the uterus, and in a patient under these circumstances, where the labour happens to be protracted and difficult, and the presentation is so far distant as not to be felt at all, or felt with extreme difficulty, the hand or foot has been grasped so distinctly as to convey the idea to the practitioner of the fœtus being extra-uterine, or of rupture of the uterus having taken place, the labour eventually having terminated naturally, and well in every respect.

"The section of the unimpregnated uterus displays an unoccupied cavity, communicating by an open mouth with the vagina below, having, therefore, properly speaking, no CONTENTS; while the gravid contains the *membrana decidua,* and the *ovum;* which latter consists of the *chorion,* the *amnion,* the *liquor amnii,* the *placenta,* the *funis umbilicalis,* the *fœtus,* and, in an early stage of pregnancy, the *vesicula umbilicalis.*

On opening the gravid uterus, besides the spongy character of its structure just adverted to, and the large size of its vessels, (which have acquired such a magnitude, that the veins have the term sinuses applied to them,) its thickness must necessarily become an object of observation. This varies considerably in different individuals; the substance is generally rather thicker than in the unimpregnated state; and in all instances the fibres are more apparent." 81.

*Membrana Decidua or Caduca.*—These names were given to the membrane by Dr. Hunter (who was the first to demonstrate its two laminæ) in consequence of its being shed from the uterus after labour, with other discharges. He also called the outer layer the *decidua vera,* and the inner *decidua reflexa.* We prefer the terms employed by Dr. R. Lee, *decidua uteri* and *decidua ovuli;* because their adoption merely describes the situation and connexion of the two laminæ, and does not involve any theory as to the formation of the inner layer, about which there is still considerable doubt. Of late it has qeen described by Chaussier under the title *epichorion*, from επι, above, and χωριον, the external ovular membrane; by Dutrochet *epione*, from επι, and ὠὸν the ovum: by Breschet *perione*, from περι around, and ὠὸν: and by Velpeau *anhiste*, from ἀ priv, and ιστος, a web. Velpeau uses this term to signify an inorganic substance, since he denies the organization of the membrane at any period of pregnancy.

The deciduous membrane is divisible into two layers, both together being not thicker than the nail, and is flocculent on that face which is attached to the uterus, smooth and plane on the one next the ovum; so glossy, indeed, that it might be supposed to possess serous properties.

In the early part of pregnancy, the two layers are separated from each other, especially towards the fundus uteri, by a quantity of red coloured fluid, partly serous, and partly half coagulated, to which *Breschet*, who designates it sero-albuminous, has given the name hydroperione. As gestation advances, this fluid is gradually absorbed, and the two laminæ come into close contact at every point, except where the placenta intervenes between them. This membrane is a product of the uterus, and does not originate in the ovum.

Hunter called the internal layer the decidua reflexa. Its value appears to be principally if not entirely confined to the first five weeks of pregnancy.

It is subservient both to the nutrition of the embryo, and to the preservation of its vitality; and thus, before the elaboration of the placenta, it seems to perform for the new being, functions analogous to those which, in an after stage, are carried on by the placenta itself.

Chorion—is a constituent part of the ovum from the remotest period of conception, because in extra-uterine pregnancy we find it, not in the uterus, as the deciduous membrane is, but enclosing the embryo itself. It possesses no blood-vessels evident to the naked eye; but we cannot deny its vascularity, since it is subject to disease, and in many of the mammalia may be readily injected.

Within the chorion is the amnion, another very thin, transparent, and tough membrane, in structure and appearance so similar to the chorion, that it is almost impossible to distinguish the one from the other. It is destitute of coloured vessels, but it too must possess vascularity; because, like its twin sister, it becomes thickened by disease, and because it enjoys in an eminent degree the power of secretion. Its use is exactly analogous to that of the chorion, so far as affording a covering to the ovum is concerned; but it performs an additional distinct function in the secretion of the liquor amnii.

The relative proportions between the quantity of liquor amnii and the size of the embryo differs much at different stages of pregnancy, being considerably greater in the earlier periods, and less at the advanced stage. Thus, when the embryo is scarcely visible to the naked eye, there is from half a drachm to a drachm of water collected within the membranes.

The origin of this fluid, liquor amnii, has given rise to much controversy. It is now generally regarded as a secretion or exudation from the inner surface of the amnion, supplied by innumerable colourless vessels, which ramify on that membrane.

*Placenta.*—Of the fœtal appendages—all of them highly essential towards the well-being of the ovum, either at the early or more advanced period of intra-uterine life—the placenta is perhaps the most important; —the medium of communication between the mother and her infant;—the organ through whose means life is sustained, nourishment supplied, and growth perfected.

"*Twin placentæ.*—In plural gestation a separate placenta, a separate funis, a distinct set of fœtal membranes, and a distinct quantity of liquor amnii, are formed for each child. The placentæ are commonly joined together at their edge, and when regarded on the maternal face, they have the appearance of a single organ. But the vessels of the one do not anastomose with those of the other; —the circulations are perfectly independent; so that the blood of one child does not pass into the system of its brother. One of the twins may, therefore, still live after the other has died;—one may be healthy while the other is the subject of disease.

It occasionally happens, indeed, that a communication exists between the vascular systems of the two children, though they are both enveloped in separate membranes; and it has been also, though very seldom, remarked, that both were wrapped up in the same bag of membranes; and that the funis, having arisen by one branch from the single placenta, has split into two divisions to supply each fœtus." 94.

That the same set of fœtal membranes envelops twins we believe is more frequently the case than our author considers, and this circumstance has more recently been remarked in cases by Mr. Gregory Smith, Dr. Henry Davies, and Mr. Dodd, of Northampton; yet in each and all of these cases the vascular circulation of each fœtus was perfectly distinct and independent of each other.*

*Battledore placenta,* or where the umbilical cord passes into the placenta at the edge; or where the vessels divide into a number of branches before they arrive at the substance of the mass, is of importance in practice.

*The funis umbilicalis, umbilical cord, or navel-string,* varies much in length; in some instances not exceeding six or seven inches; in others being more than five feet. Its average length may be regarded as from eighteen to twenty-four inches. It varies also in thickness, and this depends on the larger or smaller quantity of a viscid semi-transparent gelatinous matter, —the gelatine of Wharton—contained in cells, which constitutes the principal part of the thickness of the cord. These cells do not communicate with each other freely.

The funis gives a passage to three blood-vessels—two umbilical arteries and one umbilical vein. The arteries are longer than the vein, being considerably more tortuous; and they generally continue their course in a spiral direction, running round the vein; in the majority of cases being twisted from the left to the right.

The vein is much greater in its calibre than the two arteries together; but as the latter vessels are perhaps twice the length of the vein or more, the quantity of blood actually contained in the two arteries at one time may be nearly the same as in the vein.

The vein possesses no valves; and the arteries do not communicate

* See Medical Gazette, May, 1841.

with each other until they reach the placenta; when one generally sends off a large transverse branch to the other. The arteries carry adulterated blood from the body of the fœtus to the placenta, and have a very strong pulsation; the vein carries back again to the fœtus, pure blood imbued with the principles of both vitality and nourishment. In some respects, then, these canals may be likened to the pulmonary vessels; but the umbilical vein, by transmitting the means of growth, as well as of the continuance of vitality, performs an office superior in value to the pulmonary veins, which give passage to fluid fraught with the principles of life alone.

When the embryo is first visible, in the earlier weeks of utero-gestation, we see nothing like a funis umbilicalis; but the newly-formed being is attached by its abdomen directly to the amnion.

*Urachus.*—In the quadruped, besides the blood-vessels, there is another pervious duct running along the funis, called the Urachus. In the human subject there is no duct; but an impervious cord runs up from the fundus of the bladder, and is lost at the umbilicus.

The *Vesicula Umbilicalis*, or *Vesicula Alba*, constitutes also a part of the ovum in its early stage. It is a small sac, not larger at its greatest magnitude than a pea or swan-shot, situated between the amnion and chorion, possessing a pellucid coat, and enclosing a small quantity of viscid transparent fluid, whitish, or more generally rather of an amber colour.

*The Fœtus.*—At the end of gestation the fœtus ordinarily measures about twenty inches from the crown of the head to the heel, and weighs nearly seven pounds: but there is an amazing difference in both these respects, particularly the latter; and the size is influenced by circumstances not very easily explained. Generally speaking, males weigh more than females by one or two ounces, and are longer by a third or half an inch. Some children at full time have been known to weigh even less than five pounds; while many cases are on record where the weight exceeded double the average. Some to the extent of nearly eighteen pounds, of which weight we have seen one.

The position of the fœtus is well described and depicted.

*Development of the Uterus.*—The uterus is constantly enlarging during the whole term of gestation, and its increase corresponds with that of the ovum; so that its growth towards the close of pregnancy is comparatively greater from week to week than at any other period. The fundus and body are first evolved: and the neck does not begin to expand until five full months have passed.

The parietes do not become thinner as the uterus grows, but in many instances absolutely thicker. They are not distended by their contents as a bladder might be blown up; and the cavity is never so completely filled, but that it would hold somewhat more than it contains. The enlargement is dependent on a process of healthy evolution.

"Great as is the increase of the womb in its general bulk, the blood-vessels undergo an enlargement even far more considerable in proportion; and this is explained by the fact that they have not only to nourish the parietes, but also to supply the wants of the growing fœtus. It is this circumstance which renders the texture of the gravid womb so loose and ductile: and the amazing diameter they have acquired before labour commences, most readily accounts for the violent hemorrhages that not unfrequently attend on parturition.

This alteration in the size of the blood-vessels mainly contributes to the increase of the uterine parietes; there is nevertheless an additional quantity of both cellular and fibrous matter secreted, as the evolution proceeds. The nerves and absorbents also partake of the general enlargement; though not to so great a degree as the blood-vessels." 104.

Dr. Robert Lee in his anatomy of the nerves of the uterus remarks, Dr. William Hunter was the first anatomist who examined the nerves of the gravid uterus, and who says, I cannot take upon me to say—what change happens to the system of uterine nerves from utero-gestation, but I suspect them to be enlarged in some proportion, as the vessels are. Mr. J. Hunter who denies that the nerves of the uterus increase in the smallest degree during pregnancy, has left no preparations to demonstrate the accuracy of his statement—thinks that the intense pain and violent periodical contractions do not depend on the nerves, but on some incomprehensible vis insita on the organ. Dr. R. Lee, after quoting his own excellent dissections so well portrayed in his plates, says, these dissections prove that the human unimpregnated uterus possesses a great system of nerves, which enlarges with the coats, blood-vessels, and absorbents during pregnancy, and which returns after parturition to its original condition before conception takes place. It is chiefly by the influence of these nerves, that the uterus performs the varied functions of menstruation, conception, and parturition, and it is solely by their means that the whole fabric of the nervous system sympathises with the different morbid affections of the uterus. If these nerves of the uterus could not be demonstrated to exist, its physiology and pathology would be completely inexplicable.

Dr. Ramsbotham describes well the alteration in the relative situation of the uterus,* both with respect to itself and its peritoneum, on the adjacent parts, and says the peritoneal covering is of necessity greatly extended in surface; and this depends partly on the formation of new membrane, a fresh secretion,—partly on its allowing itself to be stretched out in every direction, (for it is highly elastic, as is shown in the variations of contraction and distention which the stomach, intestines, and bladder, are constantly undergoing, and in the descent of that portion of the membrane which constitutes the sac in hernia;)—and partly on the layers of the broad ligaments splitting, and receiving the sides of the uterus between their folds; and plates 19, 30, 31, and 32 are excellent.

### LABOUR OR PARTURITION.

These simple terms designate a very complicated process, embracing the dilatation of the passages, as well as the expulsion of the ovum.

The principal agent in labour is the uterus itself; but it is much assisted in its action by the contraction of the abdominal muscles, and probably also of the diaphragm.

Under labour the fœtus is perfectly passive: so that a dead child is expelled, generally speaking, nearly with the same ease as a living one.

* This is well portrayed in Meygner's plates.

The action of the uterus is perfectly involuntary, and consists in a contraction of the fibres embedded in its structure, which indeed form its peculiar parenchyma.

But the auxiliary muscles which assist the uterus in its contractions are in a great degree voluntary; so that labour may be said to consist of a mixed action; partly of a voluntary, but principally of an involuntary character: for the aid which the woman contributes by the exertion of her own will is not to be compared to the propelling power of the uterus, which is entirely independent of her control.

The *general features* of labour are the same in all cases, but there is an infinite diversity in the details.

After enumerating the symptoms of labour, Dr. R. says,—But of all the symptoms announcing the access of labour, pain is the most prominent.

Pain has three sources—one dependent on the simple action which, like the spasmodic contraction of muscles, is attended with suffering—another, that of opposed propulsion—and the third, that of distention of the passages.

False pains are asserted to be more frequently met with in primary pregnancies than afterwards—our own experience would lead us to doubt this, and among the causes of false pains we think might be added the gravitation of the uterus into the brim of the pelvis—where it gets jammed or wedged as it were.

Many a day and night have been spent in anxious watching over a patient, to the great inconvenience of the practitioner, to the destruction of his rest and health, and perhaps to the detriment of his professional character, when there was not the slightest necessity for such close attendance, simply because the patient would not acquiesce in the requisite examination being made.

We should say that no practitioner should remain with a patient under these circumstances—he should state to her firmly and civilly that an examination is important, either for her comfort, that he may be enabled to assure her that appearances are all favourable,—or in the event of there being anything untoward in the case requiring assistance (as preternatural presentation of the upper extremity) that he may be prepared to give it his immediate attention—should the patient then not submit to the necessary examination, he should, with all the kind consideration a patient so situated is entitled to, say that, as she is not sufficiently ill to require his attendance, he will go home, or to visit his other patients, as the case may be, and will return to her when sent for; for, by remaining with the patient he becomes responsible for the well-conducting of the case, and how can he do so in one of the progress of which he knows nothing about. If our memory fails not, the late Dr. Rigby, in his work on uterine hæmorrhage, mentions the case of a patient who would not admit of interference till it was too late to turn the fœtus with advantage, and she died.

*Classification.*—For practical purposes labours may be conveniently divided into—1st. *Natural.*—2nd. *Difficult.*—3rd. *Preternatural.*—4th. *Complex.*

We believe, for all practical purposes, this division is one of the best that can be made. Dr. R. enters very largely into the consideration of the division made by other authors, to which we refer the reader.

Labour is described as consisting of three stages: the first terminating with the opening of the os uteri to its full extent, the rupture of the membranes, and the evacuation of the liquor amnii; the second, with the birth of the fœtus; and the third with the expulsion of the placenta. We might with some show of reason add a fourth stage, considering that to end with the complete closure of the uterine vessels, and the stoppage of every chance of hæmorrhage: but as this last might continue throughout the whole puerperal month, or longer, it may be as well to follow the more ordinary usage, and to regard labour as terminated on the removal of the placenta.

*Much knowledge must be acquired* during the first vaginal examination: it is, first, whether the woman be pregnant; secondly, if she be in labour; thirdly, whether the membranes have ruptured, or are still entire; fourthly, how the child is presenting; fifthly, how far the labour is advanced; and sixthly, the state of the os uteri, vagina, and perineum, in regard to their distensibility; we might add seventhly, the dimensions of the pelvis.

"The object of covering the finger with some oily substance before making an examination, is two-fold: partly because the lubrication assists its introduction; but partly also to diminish the chance of inoculation with morbid matter, should the patient be labouring under any venereal affection. Three of my intimate medical friends have suffered most severely from secondary symptoms of syphilis communicated in this manner; and five different midwives of the Royal Maternity Charity have been the subjects of the same disease, contracted through an abrasion of the cuticle, while in attendance on women in labour. These are grievous accidents, and no means should be left unused, by which such a serious consequence may be avoided. If, unfortunately, a suspicious-looking sore should make its appearance on the finger, all obstetric duties must be abandoned until after it is healed; for another woman may be infected from the contact of an open chancre on the hand of the medical practitioner." 157.

We recommend this note to attentive observation.

With regard to aperients after delivery, we have deemed it preferable to direct an enema to be administered in due time after the *second dose*—as where a repetition of doses has been persevered in, injurious purgation has often been the result; but we must not be understood as by any means allowing an enema to supersede the exhibition of an aperient.

We strongly recommend this division of symptoms—progress, mechanism, management, and after-treatment of natural labour to the attentive perusal of the student in particular; for, without an accurate knowledge of all the circumstances connected with that state, no man can practice with comfort to himself or benefit to his patient, neither can he be adequate to the management of any untoward case that may come under his care. For this reason, also, we consider that to practice well in particular or difficult cases, a man should be an universal practitioner in midwifery.

*Difficult Labour.*—The second class of labours, DIFFICULT or LABORIOUS, embraces two orders, *lingering* and *instrumental*.

We sometimes hear of a woman being in uninterrupted labour a week, ten days, a fortnight, or even longer. Such an idea is perfectly absurd:

the powers of the system could not bear up against the exertion of labour for so protracted a period.

Such cases, then, of reputed prolonged parturition, are dependent on false, irritable, spasmodic pains, situated in some other part of the body; by which, as we have already learned, women are frequently harassed towards the close of gestation; but which are perfectly unconnected with, and independent of, contraction in the uterine fibres.

In estimating lingering labours, we calculate from the first commencement of true uterine action; but in estimating the length of labour, in reference to the patient's strength, and its effects on her system, we principally take into consideration the time that has elapsed since the membranes broke; for it is reasonable to infer that no great exertion has been sustained—consequently that little or no exhaustion has appeared; and particularly, that scarce any injurious pressure can have taken place on the soft parts within the pelvis, while the membranous cyst remained entire; provided there be an ordinary quantity of liquor amnii.

*Insufficient Uterine Action.*—After speaking of different means applicable for rousing the uterine energies, Dr. R. goes on to say:—

"Various specific medicines have been recommended at different times, to increase the parturient throes, and facilitate the child's birth; but I believe that the whole of these substances, one only excepted, act upon the womb through the excitement induced in the arterial system. They first stimulate the nervous, then the arterial, and through the medium of those systems, the uterus. Almost the only medicine now used as an uterine excitant, is the *ergot of rye:* and I have no hesitation in declaring my opinion that its action is specific, and that the uterus is not affected through any disturbance first set up in the arterial system." 217.

"This drug has been exhibited in various forms, chiefly in powder, infusion, decoction and tincture. The two first are in my opinion the best modes. If given in fine powder, about twenty grains is the proper dose; but I am myself generally in the habit of exhibiting it in infusion. Two drachms may be infused in four or six ounces of boiling water for twenty minutes; a fourth part of the strained liquor should be given at a time, and under labour the dose may be repeated every quarter of an hour, until either its action becomes apparent, or the whole is taken: for I consider it useless to persevere with the medicine, if the quantity mentioned produces no effect. I have found that if the infusion be allowed to stand much longer than the time I have specified, it acquires a nauseating property which greatly distresses the stomach. Desgranges used only the black cortical part, in which he considered its active principle to reside: he gave it in doses of four or six grains, which he found as efficacious as thirty grains of the whole powder. Villeneuve administered it in *lavements;* and he considers this the most efficacious means of employing it, provided there be present much irritability of the stomach." 219.

Dr. R. is not aware of the ergot ever having produced any pernicious effect on the system, and some individuals are not affected at all by it. Its effects upon the uterus in labour are often speedy, powerful, and astonishing. Its action mostly commences within fifteen or twenty minutes after its exhibition, and the character of the pains is peculiar. When the ergot has obtained a full power over the system, the uterus often acts without any decided intermission for many minutes together; there being only a slight remission observed—no interval of perfect ease.

As the ergot undoubtedly possesses such a strong influence over the uterine system, it is evident that, if exhibited improperly, it is likely to do great injury.

It ought never to be exhibited where there exists any disproportion between the fœtal head and pelvic cavity. Where there is any rigidity of the parts, or in malposition of the head, or in preternatural presentation, or in first children generally.

" It must only be given in cases where the sole cause of delay is a torpid or feeble state of uterine action : or where it is desirable to terminate the labour speedily,—and that too by means of the natural powers, in consequence of hæmorrhage. I have found it very useful in accidental hæmorrhage after the membranes have been ruptured; in loss of blood under abortion also, where it was impossible to empty the uterus by manual operation ; and where the patient would perhaps have sunk from the continuance of the bleeding."

We cannot conclude the remarks on the exhibition of the ergot of rye without entering our most solemn protest against its abuse; miserable are the effects we have witnessed from its mal-administration, and that when given by individuals in extensive practice. We earnestly recommend the perusal of the cautions laid down so well by Dr. Ramsbotham, and we implore every practitioner to consider well on the case and of the effects of the medicine before he exhibits it. We have little hesitation in saying, after a pretty extensive experience both in private practice and in public institutions—that more harm than good has been done by the ergot. And as to its superseding the judicious application of the forceps except in cases of simple arrest from defect of pain, we believe it quite out of the question. We nevertheless consider the ergot of rye a most useful and powerful agent in very many cases, when used with due consideration and caution.

Among other adjuvants in lingering labour, Dr. R. recommends—stimulating enemas, warmth externally, also pressure and friction are found to possess greater power over the uterine fibres than warmth externally applied. The pressure of a bandage, or the hand, will often excite the uterus to increased action both before and after the birth of the child.

He also mentions the advantage of change of position, walking, &c., and of the possible utility of electrical shocks, and cautions against the early rupture of the membranes—or rubbing the os uteri.

The second cause of lingering labour embraces those cases in which the uterus is acting powerfully and energetically, but where there is a want of due and proportionate space in the passages for the ready exit of the child : and of these causes, *distortion of the pelvic bones*, as being one of the most frequent and difficult, claims our first attention.

Of the Cæsarian operation, he says, if performed at all, it ought to be performed early—and mentions the works of Mr. Barlow and the operation of Mr. Knowles. We think much useful information will be found in the work of Dr. Hull of Manchester.

We have never seen a case of distorted pelvis requiring this operation; nor, except in large manufacturing towns, where girls from their birth are distorted, we believe, in this country do such cases occur. The Doctor's remarks on this subject will be perused with advantage.

The second variety of lingering labour, or jammed head, will be noticed hereafter.

The third variety of lingering labour is the *presence of tumors* in the cavity of the pelvis. The tumors which may impede parturition vary exceedingly in their nature, consistency, and size; sometimes they possess the solidity of bone itself; at others, their contents are of the most fluid character. According to their size and unyielding nature, will be the difficulty which they occasion.

After mentioning exostoses, scirrhous or dropsical ovary, scirrhous glands, polypi, with their diagnosis and treatment, we come to descent of the bladder, which sometimes prolapses before the head.

Much embarrassment, and no small danger, may be the consequence of the case being overlooked or mistaken. This usually occurs in the early period of labour, before the head has engaged in the pelvic cavity, and depends on pressure exerted by the descending head upon the fundus, or middle portion of the organ, at a time when it is partially distended with urine.

The symptoms attendant on prolapsed bladder are very distressing; and are to be relieved by the introduction of the catheter. Our author adds—keeping in mind the possibility of the bladder being pressed downwards as described, it is an essential duty never to puncture any fluctuating pelvic tumor, without being first assured that it is not vesical, by the removal of the urine by means of the catheter.

A fourth cause of lingering labour consists in rigidity of the soft parts: under this head are some valuable remarks and details of some interesting cases. Where the rigidity is the result of disease, as common rigidity, independent of diseased structure, is more usually met with—we think it should have taken precedence. With regard to the utility of abstraction of blood and the administration of opiates in rigidity of the os uteri, we have found the combination of the two remedies very valuable; and, where it was of importance to produce the effect with as little loss of blood as could be to our patient, we have bled the patient while she is standing if possible, otherwise sitting upright—by this mode fainting is more speedily induced—this followed by the exhibition of a large dose of tinct. opii, (or an opiate enema as may be best suited to the case); by these means the parts become relaxed, and the pains completely suspended, and perhaps succeeded by some tranquil sleep, from which, when the patient arouses refreshed, the parts often give way readily to the renewed pains. We have found this practice eminently useful, in preternatural presentations of the upper extremities, combined with violent labour-pains, when it was extremely hazardous or impossible to turn; and also in cases where the secale cornutum had been injudiciously administered: for the most part, we prefer the opiate given by the mouth, as the administration itself of the enema excites local action, or the enema is not retained long enough to take effect.

Enemas of tobacco, common enemas, application of belladonna, mucilaginous injections, warm bath, nauseating doses of antimony, emetics, are severally treated of. Meddlesome practice and other injudicious interference, and particularly the mal-administration of the ergot of rye, are strongly deprecated, and some additional milder means spoken of: one principal use, however, of these latter means is to gain time, so as to allow the natural powers an opportunity of exerting themselves efficiently, and at the same time to convince the woman that our mind is directed towards affording her relief, and thus, both hope and confidence are inspired; and perhaps greater advantage gained, than by any of the more positively useful attributes of these applications.

The observations on cicatrix of the vagina as a cause of lingering labour are worthy of attention.

*Lingering Labour from Causes referable to the Ovum.*—Preternatural toughness of the membranes is by no means a usual cause of lingering labour; it is by far more common for a premature rupture of the membranes to produce a protraction of the process. If, however, the membranes are exceedingly strong, as they occasionally are, with due caution and considerations which are laid down, they may be ruptured.

*Head preternaturally enlarged from healthy formation, monstrosity or disease (particularly congenital hydrocephalus).*—We most earnestly recommend to the attention of the reader the whole of this division of the causes of lingering labour, and also that caused by distended bladder. Most miserable have been the results that we have been witness of to patients under these circumstances, as rupture of the uterus—death immediately after delivery—or what is almost worse than death, a life of torment from sloughing of the parts, cysto-vaginal fistula, &c.—when we believe, in one and all the cases, the event might have been prevented by timely assistance; for in all these cases we believe we are for the most part able to save our patients. We may therefore forcibly express, in the words of our author, with regard to enlarged head:—

When we have ascertained that nature is unable to overcome the difficulty except at a great expenditure of power, conjoined with imminent risk to the woman's life, we are fully warranted in having recourse to perforation more early than if the child were healthy, that the fluid may be evacuated, and an opportunity afforded to the bones to collapse; the case will then most probably be terminated by the contractions of the uterus alone.

*Instrumental Labour.*—Although in skilful, and especially discriminating hands, obstetric instruments must be regarded as great blessings to the suffering sex, yet it is a question with some practical men, whether by their unnecessary and improper use they have not produced on the whole more injury than good. But we would endeavour deeply to impress upon the mind of the young practitioner that urgent necessity alone will warrant him in taking an obstetric instrument in hand: and that when a choice is allowed him, he should leave nature to accomplish her own purpose, provided, indeed, he can with safety trust her.

The whole of the directions as here laid down, are deserving of great attention. With regard to the position of the patient, it is of great importance to the success of the operation;—we have found it of great advantage, in order to keep the patient in the position directed, to have a female attendant set on the bed with her back close to the patient, and her feet towards or pressed against the opposite bed-post—this person may also separate the patients knees when required; the patient may pass her arms round her if she chooses: these arrangements steady the patient well and preserve her from jerking away at the moment when it may be of considerable importance for the safety of the soft structures.

The variety of the form of the short forceps the practitioner may prefer (if judiciously used) is not of serious consideration. Our author remarks on the comparative safety of the one over the other; for our own part we prefer decidedly those, with a convex edge to each blade, of Osborn. The addition of a pin in the *middle* of the one handle, which is received into a deep groove of the other, and which keeps the handles in apposition while not acting during the intervals of pain, is an improvement. We have reason to believe that the forceps are still too frequently and injudiciously applied. Where the head is so jammed as to render the preservation of the infant's life impossible, we have no hesitation in saying, that the operation of craniotomy, horrible as it is, is the safer and more appropriate operation: for we would ask, what advantage is it to the patient, after all the suffering she has undergone, to have a dead child, with her parts contused (if not lacerated) and the consequences resulting from it. No practitioner with moderate care ought, by the operation of craniotomy, to inflict the least injury on the mother.

With regard to the time of using the short forceps in cases where they are required and applicable, we think it infinitely safer to apply them early than to procrastinate until the chance of preserving the infant is doubtful, and of the parts being contused certain. Dr. Hamilton (Practical Observations, Part II.) says, " cases requiring the use of the forceps occur so seldom when the first stage of labour is properly conducted, that, in the course of forty-eight years' practice, he has only had occasion to employ that instrument thirty-three times, where he had charge of patients from the beginning." He further says, no intelligent practitioner would wait in cases where the labour throes cease to have any influence in advancing the delivery, if the head of the infant be within reach of the forceps, till there be " heat or tenderness of the passages," and still less till " the patient's strength be much exhausted." Again, the principle by which he has always been directed in cases of protracted labours, to which he has been called by other practitioners, has uniformly been to consider the state of the mother principally, but not exclusively. Thus, if immediate delivery be required, he always ascertains whether the use of the forceps be safe, for if there be any evidence that there has been injurious pressure upon the passages, he considers it to be unwarrantable to employ that instrument.

The extraordinary difference which prevails in the practice of obstetricians of different countries in the frequency of using the short forceps is forcibly pointed out in a paper by Dr. R. Lee, in the Medical Gazette, Vol. 24, Sept. 1839, p. 827-8.* The long forceps we consider so hazard-

* While penning this note, a midwife belonging to one of the most extensive lying-in charities in London, and who has also a very considerable private practice—upwards of ten years came in; we asked her how many forceps cases she had seen? She answered—none!

ous an instrument that no practitioner, unless he was very much accustomed to operative midwifery, would venture to use them. We forbear saying more of them than cautioning against their abuse. (We should like very much to learn how many infants' lives have been preserved by them without materially injuring the patient's structures). We are disposed to recommend the guarded crochet, for our left hand has more than once suffered from slipping of the crotchet, with due attention to all the rules laid down by Dr. R., and we also use a craniotomy forceps—but rather lighter than the one recommended by Dr. Holmes, and which we find most useful.

*Induction of Premature Labour.*—The arguments on this subject, pro and con, are well defined. We have tried all the plans laid down by Dr. R. with variable success, but for the last ten years we have for the most part preferred puncturing the membranes high up, and letting off a small quantity of liquor amnii, with an instrument of Dr. Holmes's somewhat modified, consisting of a canula slightly curved at the end, having a wooden handle, the whole about thirteen inches long, and containing a concealed flexible trocar, moved by a spring at the end of the handle. The patient having had her bowels well emptied by a brisk purgative or two, —the os uteri being somewhat dilated by the finger—the instrument, guided by the finger, is carefully introduced as far within the uterus (mostly at the posterior part) as it conveniently can be, between the uterus and membranes; having ascertained that the point of the instrument is opposed to the membranous bag of fluid, the handle is carried in the opposite direction and the spring pressed on, by which the trocar is projected out, and the membranes pierced, a moderate quantity of fluid (liquor amnii,) from ℨss. to ℨj. is allowed gradually to escape by the side of the instrument, which is then withdrawn; the patient is desired to go about as usual, or rather to take a walk, the liquor amnii continues to dribble away, uterine action is at length induced, and the majority of children are born within seventy-two hours from the time of puncturing the membranes.

Of the advantages of dilating the os uteri with sponge and lint, we have had no experience.

The greater number of women, as Dr. R. observes, are not able to nurse their children, but we have had several patients who have done so very efficiently.

We most fully agree with the Dr. that this operation should never be performed (unless from very cogent reasons, and confirmed by a consultation) in a first pregnancy.

In difficult breech presentations Dr. R. objects to the inapplicability of the forceps, says, "the instrument not being made for the breech, but for a more globular body, does not fit that part, and is liable to slip, to the great hazard of the mother's structures. The only way, indeed, by which we can cause the blades to keep their hold at all, is by squeezing the handles firmly together, so that the points may take a deep nip upon the fœtal body; and the youngest student in anatomy would at once call to memory important organs likely to suffer severely from this rude pressure." Either of the three modes, the use of the finger, the application of a handkerchief, or of the blunt hook, if the former two are not applicable, is to be preferred; and in this we cordially agree.

In transverse presentation, after describing very accurately the diagnostic symptoms of the different varieties, and the modes of affording assistance, directions are given for the position of the patient (p. 433) which is of the utmost importance to the satisfactory performance of the operation, (we have seen very able practitioners fail from inattention to this,) and for the necessity of baring the arm is insisted on, (which in some works we have been astonished to see as not required,) turning with the left hand is advised, and, among other reasons, that the right may be applied to steady the uterine tumour. It is no doubt of great advantage to be able to use either hand as may be requisite, but of the steadying of the tumour with the right applied on the abdomen we are somewhat sceptical—we believe that the patient is better steadied in the manner we have described for delivering with the short forceps. Before drawing down the part of which we have hold, we are most forcibly and properly cautioned to take care that we have a foot and not the hand. He also says, I think it highly desirable that both feet should be taken hold of, if they lie together and can be commanded by the same effort, because the evolution is so much more easily accomplished when both are brought down; but if one only be obtained, it is neither necessary nor proper that we should spend time, and inconvenience our patient, by searching for the other.

Mr. Barlow's note is a very useful precaution—that "on all occasions when the introduction of the hand into the uterus is required, it is necessary that the contents of the bladder and rectum be previously emptied."

Notwithstanding the Doctor's dissent, we have frequently in arm-presentations (where it has been impossible, by any warrantable exertion, to introduce the hand into the uterus, from its strong contraction and energetic pains) succeeded after a copious bleeding, and a large dose of tinct. opii, in bringing down the feet; and, unless other symptoms required it, we have left them there till the uterine action was restored, and then with very moderate assistance the infant has been expelled.

We have now and then in arm presentations, where the head was in the vicinity, by pressing up the presenting part, enabled the head to come down; and by keeping up the presenting part with the points of the fingers till the head was fully engaged, the labour has terminated naturally, and the infant been born living.

Dr. R. has described faithfully and accurately, with the aid of the plates, the symptoms and management of the breech and transverse presentations, cautioning against meddlesome practice on the one hand, and recommending, on the other, energetic practice where such was requisite. This division of the subject is written with a masterly hand.

*Complex Labour.*—Hæmorrhage during labour is by far the most frequent source of danger to the lying-in woman; and since it is so common in occurrence, so alarming in its nature, and fatal in its effects, this accident calls for the most anxious and serious attention. All profuse hæmorrhages during parturition, and towards the close of gestation, are to be regarded as originating in a partial detachment of the placenta from the uterine surface, and the consequent opening of a certain number of vascular orifices. It is surprising to notice how slight a degree of depression will follow an excessive flooding in some women: and how small a discharge will destroy others. We have known two women die from the eruption of scarcely a pint of blood; and we have seen others recover perfectly when they have suffered the loss of some quarts. Hence we are to be influenced in the immediate measures we put in practice more by the effect on the constitution than by the apparent quantity of blood lost.

Dr. R. considers the various means used for arresting hæmorrhage generally, and then the treatment more particularly applicable to uterine hæmorrhage during labour, which he divides into *unavoidable* and accidental hæmorrhage. In the management of unavoidable or placental presentation, absolute and uninterrupted quietude in the horizontal posture, and on a hard bed, must be forcibly enjoined, and an anti-hæmorrhagic regimen prescribed; she must breathe a cold atmosphere; be but lightly covered; her diet must principally consist of nutritious fluids—cold and acid drinks may be given almost *ad libitum*, and ices may be allowed, unless they produce intestinal pain or shivering: every thing stimulating, both alcoholic or of any other nature, must be strongly forbidden. The mineral acids, under such a case, may be usefully employed; some gentle aperient will be required, and the acidulated infusion of roses, with small doses of sulphate of magnesia, is perhaps the pleasantest, and, at the same time, as efficacious a medicine, as any that can be exhibited. We must avoid a constipated state of bowels; because the straining necessary for the passage of hardened fæces may dislodge the coagula collected over the exposed vessels, and produce a return of the flooding. We must equally, also, avoid violent purging, lest the frequent evacuation of the rectum should occasion a like disaster. A cold enema may be administered daily, which will probably act beneficially in two ways,—both by clearing the rectum, and restraining the hæmorrhagic tendency.

The Doctor has little faith in the power of opium to suppress hæmorrhage—and thinks that the milder narcotics, as henbane and hemlock, are equally useful in tranquillizing the excited state of the nervous system—without diminishing the uterine energy. But, the Doctor says, a time will arrive when the features of the case will be changed; and the delivery of the infant must be resorted to—the reasons for which, the appropriate time and mode of performance, are fully discussed. For the purpose of controlling the hæmorrhage until the time for delivery arrives, we consider the plug a most valuable remedy, and the best material for the purpose, a soft moistened sponge pressed close to the os uteri. The advantages of this our author does not seem fully to appreciate. When the time for delivery does arrive, there is no case where, as he well says, procrastination is so prejudicial, and promptness in the execution so absolutely necessary to the patient's safety, for she is on the very brink of a precipice. We have passed our hand through the placenta repeatedly—without any of the disadvantages the Doctor enumerates—and in accordance with his reasoning, p. 482, "that in every increase of a line's diameter (in the placenta's detachment) there will be an increase of bleeding." If we are quite sure which is the nearer way to the edge of the placenta, his rule is no doubt the better one. Promptness in determination to act does not imply *hurry* in the execution. We are told, most pointedly and correctly, (p. 483,) "that our hand must be passed slowly and carefully onwards: and again, that rapid extraction of the child's body should be cautiously avoided."

Partial presentation of the placenta admits, for the most part, of the same mode of treatment as accidental hæmorrhage; nevertheless, it must not be supposed that a natural termination, though so highly desirable, will invariably follow the proceeding I have recommended: the after-conduct of the case, then, must depend on the continuance of the hæmorrhage, and the effect produced on the constitution.

*Accidental Hæmorrhage.*—It is mostly observed in accidental hæmorrhage, that, after the establishment of labour, the discharge is diminished in quantity, or wholly suspended, while the uterus is contracting; and returns more copiously in the intervals of action.

In accordance with this observation, early rupture of the membranes, for reasons fully detailed, is recommended. We do not know any obstetrical practice which is more generally satisfactory in its results, than early rupture of the membranes in labour with accidental hæmorrhage, together with a firm bandage round the abdomen, tightened (but not to girt) as the uterus decreases in bulk, and with the occasional aid of the ergot of rye.

Out of a considerable number of cases so treated, we have seen somewhere the result was fatal; and, we believe, (as Dr. Lee observes in his detail of some fatal cases among a number of successful ones,*) that, in these, death could not have been prevented by any means we possess.

Dr. R. further says—"Should the discharge continue to flow outwardly with profuseness, or should indications of internal bleeding be present—the symptoms, indeed, being those of loss of blood generally, together with a flabby and relaxed state of the uterine parietes—delivery must be had recourse to without delay, as offering the only reasonable chance of safety."

Hæmorrhages after delivery, with their treatment, and the operation of transfusion, are well discussed. On the management of the placenta we have full and excellent directions. On the treatment of retained placenta from irregular contraction, we are told—"since, then, there is so much more chance of injuring the uterus, it behoves us to be so much the more cautious in our proceedings. If there be no flooding, we may generally wait an hour from the birth of the child; and in the interval, we may administer small doses of laudanum occasionally.

Of the propriety of giving small doses of laudanum, under these circumstances, we are quite at issue with the Doctor; but in his remarks farther on, p. 526, we more fully coincide. "Our obvious indication, if we were foiled, would be to place the patient in some degree under the influence of opium, and take advantage of the earliest opportunity of its action to renew our attempts; for the longer we wait, the more difficulty we shall experience from the permanent contraction which will assuredly take place, and which we have no means, as far as I have been able to judge, of removing."

---

* Medical Gazette, Sept. 1839.

We may here state a case somewhat in accordance—but the treatment of which we do not recommend to be followed, unless from absolute necessity. We were called to a patient at mid-day who had been delivered naturally at two o'clock in the morning; fruitless efforts had been made for the delivery of the placenta by three several practitioners, and at the moment we entered the room, the patient was making a most distressing outcry from the pain inflicted by the attempts of one whom we knew to be a most adroit practitioner: the uselessness of further exertion, together with the contused state and extreme tenderness of the parts, induced us, after consulting—other minor circumstances being attended to—to give a large and influential dose of laudanum in a saline draught, and to wait patiently. Early the morning following an active aperient was given, and four hours after (the aperient not acting) a domestic enema was administered—strong uterine action returned, when the bowels acted, and a very large placenta was expelled without any further interference. The patient did well.

Convulsions is the most formidable disease puerperal women are liable to. This disease assails women of all ages and constitutions (p. 563,) still, cæteris paribus, women of the plethoric or nervous temperament are most obnoxious to their attack, and they by far more frequently accompany first labours than subsequent ones. We agree with our author (p. 564, note) that too minute nosological arrangements are bad, particularly where they do not lead to any practical difference in the treatment; but we do consider that some modification in the treatment is required where puerperal convulsions assail women of the different temperaments before mentioned.

Dr. R. considers puerperal convulsions to be exactly analogous to infantile convulsions, p. 566, (where the nervous system bears a very large proportion to the general bulk of the body), "and that they are both of them allied to apoplexy; the causes, however, acting upon the system under a highly excitable state. This view of the case, whether correct or not, is practically valuable, and will lead to the most judicious treatment." "But the affection, in my opinion, originates most frequently in some deranged state of the uterus itself, probably in its nervous system, and consists in some irritation propagated from that organ to the brain."

In the treatment, the active exertion of all our skill and energy is required—copious bleeding and other appropriate treatment, are fully descanted on, p. 575-9.

*After active* depletory means have been had recourse to, and the convulsions have apparently ceased, we think we have seen the recurrence of the paroxysm arrested by dashing the patient's face with cold water, (by a basin full out of a pail,) at the moment we observed the face portray the approach of convulsion by its becoming distorted. "Few (cases) comparatively, under good care, now terminate unfortunately; and the favourable results are to be attributed to the extent to which bleeding and other evacuant means are carried."

The bladder is recommended to be *carefully* attended to (p. 587), not only during the convulsive paroxysms, but also while a state of imperfect consciousness remains.

*Rupture of the Uterus.*—This most formidable accident usually terminates fatally. Among the causes, we think not forcibly enough stated by authors, may be mentioned congenital hydrocephalus of the fœtus, and we are therefore induced to detail two cases from this circumstance, the fatal termination of which might have been prevented. In the first, the patient had borne eleven living children—in her 12th labour she was attended by her usual midwife—her labour went on for two days, and not making any satisfactory progress, a second midwife saw her; as the head presented at the brim of the pelvis and no hæmorrhage appeared, and the parts were lax and dilatable, they, the midwives, considered it a mere tedious case; a neighbouring medical practitioner was subsequently called in, when she was dying; and she was dead before we reached her.

The late Mr. Joshua Brooks, with several medical friends, assisted at the examination. On examination per vaginam, there appeared nothing extraordinary—on laying aside the abdominal parietes, the uterus appeared full, prominent and healthy—on opening the uterus, the fœtus lay in the natural position; with considerable difficulty the fœtus was taken out, the head was so completely and tightly jammed in the brim of the pelvis, the uterus was literally worn through on one side, near the cervix, between the fœtal head and brim of the pelvis. Blood had escaped to some extent under the peritoneum into the lumbar region. The patient had an ample well-formed pelvis.

The second patient had also borne several living children. Labour had been going on for thirty-six hours—her usual medical attendant had been with her eight hours—and had gone to lay down on the sofa, she was seized with rigor—we were sent for—the head was perforated—a large quantity of water escaped—there were pains enough to propel the fœtus, and the placenta followed with moderate assistance—shortly after, the patient died. The uterus was lacerated on the side from the cervix to near the fundus—the pelvis was ample. It has occurred to us to have seen several cases of congenital hydrocephalus in labour, in some of which we have reason to believe the fatal termination was prevented by delivering the patient by the operation of craniotomy. Ample and judicious rules are laid down for the detection and treatment of these cases by our author, under the divisions of lingering labour from enlarged head, and craniotomy.

*Rupture of the Bladder.*—"It appears to me that this accident must always be the effect of neglect or improper interference; it very seldom indeed, or never can occur in the hands of a careful and judicious surgeon."

Since rupture of the bladder is so universally fatal, and since it can usually be prevented if proper attention be paid, it becomes our duty, under lingering labour particularly, to keep a watchful eye over its condition; and, if it become immoderately full, to relieve it by the catheter.

We have afterwards fully treated, collapse, prolapsed navel-string—descent of the hand with the head—monsters and plural births—to this we have added an interesting and valuable appendix. The remarks and directions on fœtal animation suspended, we strongly recommend to the attentive perusal of our obstetrical brethren—as pointing out the best means, and holding out encouragement to them in their endeavours to procure resuscitation.

We do not know why, but we presume for some judicious reason, Dr. R. has omitted mentioning the symptoms of pregnancy, the treatment of maladies now and then attending its progress, and also the subject of abortion ; as we consider them forming a part of obstetrical medicine.

There are some subjects, as that on the management of the placenta, which in lectures orally delivered, cannot be too forcibly or strongly impressed, but which, in a written work, appear somewhat redundant, and might, without prejudice to its utility, be curtailed.

In concluding our review, we most earnestly recommend this work to the student, who wishes to acquire knowledge, and to the practitioner who wishes to refresh his memory, as a most faithful picture of practical midwifery ; and we can with justice say, that altogether it is one of the best books we have read on the subject of obstetrical medicine and surgery.

